# Identifying patterns of care for elderly patients with non-surgically treated stage III non-small cell lung cancer: an analysis of the national cancer database

**DOI:** 10.1186/s13014-018-1142-7

**Published:** 2018-10-05

**Authors:** Eric D Miller, James L Fisher, Karl E Haglund, John C Grecula, Meng Xu-Welliver, Erin M Bertino, Kai He, Peter G Shields, David P Carbone, Terence M Williams, Gregory A Otterson, Jose G Bazan

**Affiliations:** 1 Department of Radiation Oncology, at the Arthur G. James Cancer Hospital and Richard J. Solove Research Institute, The Ohio State University, 460 W. 10th Avenue, Columbus, OH 43210 USA; 2 College of Public Health, at the Arthur G. James Cancer Hospital and Richard J. Solove Research Institute, The Ohio State University Comprehensive Cancer Center, Columbus, OH USA; 3 Department of Internal Medicine, Division of Medical Oncology, at the Arthur G. James Cancer Hospital and Richard J. Solove Research Institute, The Ohio State University Comprehensive Cancer Center, Columbus, OH USA

**Keywords:** Non-small cell lung cancer, Stage III, Elderly, Patterns of care, Chemoradiation

## Abstract

**Background:**

To compare patterns of care for elderly patients versus non-elderly patients with non-surgically treated stage III non-small cell lung cancer (NSCLC) using the National Cancer Database (NCDB). We hypothesize that elderly patients are less likely to receive curative treatments, including concurrent chemoradiation (CCRT), compared to non-elderly patients.

**Methods:**

We identified patients from the NCDB between 2003 and 2014 with non-surgically treated stage III NSCLC. We defined elderly as ≥70 years old and non-elderly <70 years old. Treatment categories included: no treatment, palliative treatment (chemotherapy alone, radiation (RT) alone <59.4 Gy or chemoradiation (CRT) <59.4 Gy), or definitive treatment (RT alone ≥59.4 Gy or CRT ≥59.4 Gy). Differences in treatment between elderly and non-elderly were tested using the χ^2^ test.

**Results:**

We identified 57,602 elderly and 55,928 non-elderly patients. More elderly patients received no treatment (24.5% vs. 13.2%, *P <* 0.0001) and the elderly were less likely to receive definitive treatment (48.5% vs. 56.3%, *P <* 0.0001). CCRT was delivered in a significantly smaller proportion of elderly vs. non-elderly patients (66.0% vs. 78.9%, *P <* 0.0001 in patients treated with definitive intent; 32.0% vs. 44.5%, *P <* 0.0001 in patients receiving any treatment; and 24.2% vs. 38.6%, *P <* 0.0001 amongst all patients).

**Conclusions:**

In this large study of patients with non-surgically treated stage III NSCLC, elderly patients were less likely to receive any treatment or treatment with definitive intent compared to the non-elderly. The lack of use of concurrent or sequential chemotherapy in the elderly with stage III NSCLC suggests that the optimal treatment approach for this vulnerable population remains undefined.

**Electronic supplementary material:**

The online version of this article (10.1186/s13014-018-1142-7) contains supplementary material, which is available to authorized users.

## Background

Non-small cell lung cancer (NSCLC) is a disease of the elderly with over two-thirds of cases occurring in patients aged ≥65 years and an incidence rate that is >3-fold higher in patients ≥70 years compared to patients aged 60–69 and > 10-fold higher compared to patients <60 [1, 2]. Nearly one-third of newly diagnosed NSCLC presents as stage III disease [3]. The standard treatment recommendation for unresectable stage III NSCLC on the basis of randomized trials and meta-analyses is concurrent chemoradiation (CCRT) [4]. The applicability of this treatment recommendation to elderly patients has been questioned due to the limited number of elderly individuals treated on stage III NSCLC clinical trials [5–7].

While the data supporting CCRT for stage III NSCLC in the elderly are mixed [8–11], we recently demonstrated that chemoradiation (CRT), both CCRT and sequential chemoradiation (SCRT), is associated with improved outcomes when compared to radiation alone using the National Cancer Data Base (NCDB) [12]. Furthermore, results from the phase III JCOG0301 trial which enrolled elderly patients with unresectable stage III NSCLC, demonstrated improved survival with chemoradiation compared to radiation therapy alone [13]. This supports the notion that the addition of chemotherapy to radiation results in superior survival compared to radiation alone in the elderly population. In the current study, we set to fully characterize and compare patterns of care in elderly (≥70 years old) vs. non-elderly (<70 years old) patients with non-surgically treated stage III NSCLC. We hypothesized that elderly patients were less likely to receive any treatment or curative intent treatment compared to younger patients.

## Methods

The NCDB, a combined effort of the Commission on Cancer (CoC) of the American College of Surgeons and the American Cancer Society, is a nationwide hospital-based database that contains de-identified hospital registry data from more than 1,500 accredited facilities and represents more than 70% of newly diagnosed cancer cases in the United States [14]. The NCDB collects data on patient demographics and comorbidities, tumor characteristics and staging details, primary therapies administered, and OS. The CoC’s NCDB and the hospitals participating in the CoC NCDB are the source of the de-identified data and have not verified and are not responsible for the statistical validity of the data analysis nor the conclusions presented in this study.

### Patient selection

**Fig. 1:**
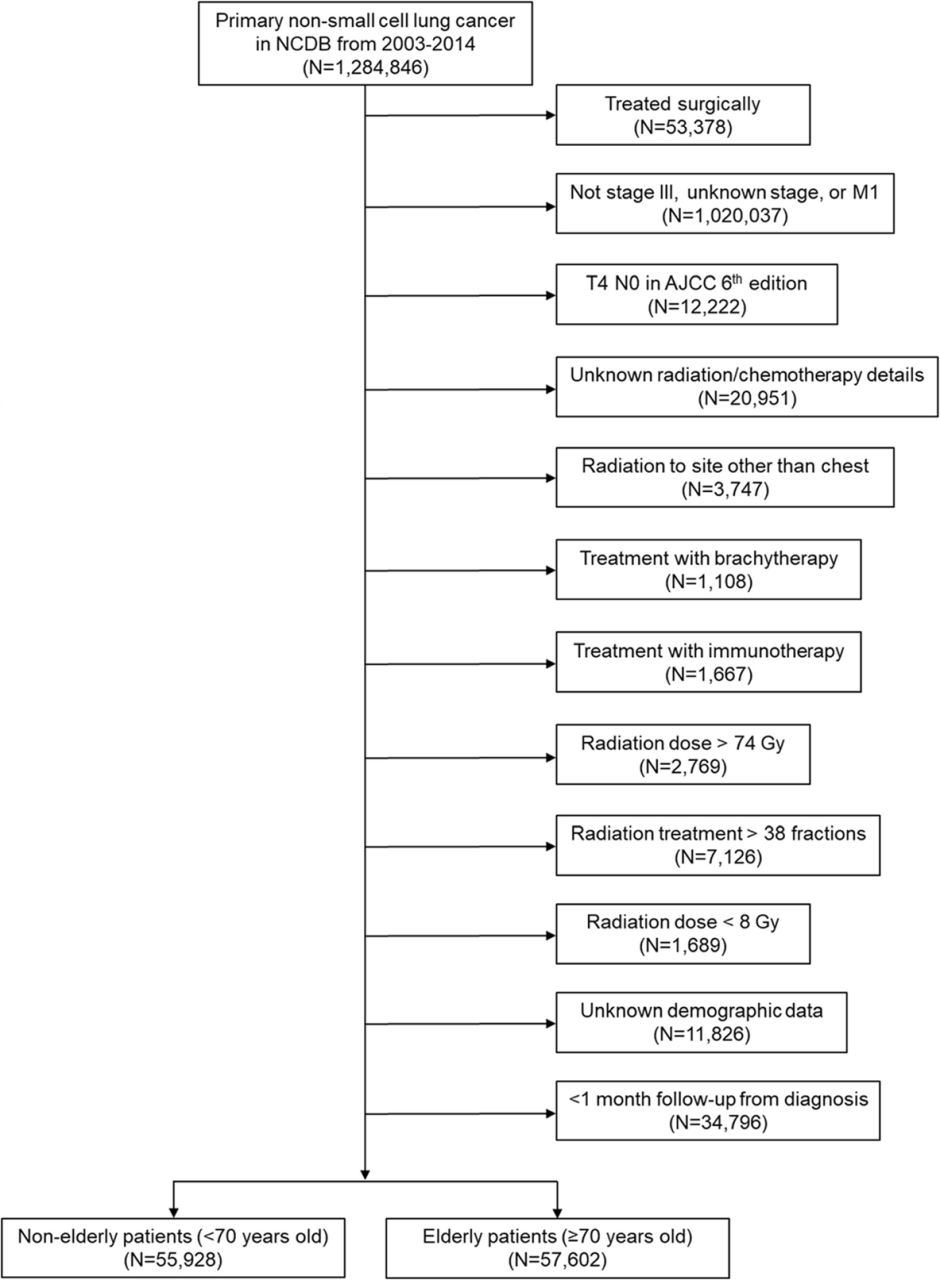
Study flow diagram for analytic cohorts. Abbreviation: NCDB, National Cancer Database

Patients diagnosed with stage III NSCLC from 2003 to 2014 were collected from the NCDB participant user file with additional inclusion and exclusion criteria summarized in Fig. 1. We defined elderly as patients ≥70 years old, as used previously in numerous studies [11, 13, 15–17] and non-elderly as <70 years old. The transition to the American Joint Committee on Cancer (AJCC) 7th edition occurred in 2010, consequently, our patient cohort consisted of a mix of patients staged using the AJCC 6th and 7th editions. We have previously characterized the patient selection criteria [12] with the exception that in this analysis, patients that received > 74 Gy were excluded.

### Treatment definitions

Treatment with palliative intent was defined as treatment with chemotherapy alone, RT alone to doses <59.4 Gy but >8 Gy, or a combination of chemotherapy and RT delivered either sequentially or concurrently where the delivered RT dose was <59.4 Gy but >8 Gy. We used 59.4 Gy (as opposed to strictly ≥60 Gy) as our cutpoint for definitive treatment based on the dose used by the Hoosier Oncology Group evaluating consolidation docetaxel after definitive chemoradiation for inoperable stage III NSCLC [18]. Patients were considered to have received concurrent CRT if chemotherapy was delivered within 30 days of the initiation of RT while sequential CRT was defined as chemotherapy delivered >30 days within the initiation of RT as defined in prior studies [12, 19].

### Study variables

We dichotomized the following baseline covariates: gender (male vs. female), race (white vs. non-white), median income (≥$48,000 vs. <$48,000), primary insurance payor (private vs. non-private), county location (metropolitan vs. urban/rural), facility type (academic vs. community/comprehensive community/integrated network programs), and clinical stage group (IIIB vs. IIIA). The Charlson-Deyo score, a measure of comorbidity was dichotomized as 0 (no comorbities) or 1 (≥1 comorbidity). Distance to the nearest facility was analyzed as a continuous variable.

### Statistical methods

The primary objective of this study was to evaluate patterns of care in elderly vs. non-elderly patients with stage III NSCLC not treated surgically. Differences in treatment modality between elderly and non-elderly patients were tested using the χ^2^ test. Logistic regression was used to identify predictors of: 1) No treatment vs. treatment, 2) palliative treatment vs. definitive treatment, and 3) RT alone vs. CRT. Variables with *P* ≤0.10 on univariate analysis were included in the multivariate model. All statistical analyses were performed using SAS, version 9.4 (SAS Institute Inc., Cary, NC).

## Results

### Baseline characteristics

We identified 57,602 elderly patients and 55,928 non-elderly patients that met the study criteria (Fig. 1). Patient characteristics for the entire cohort are shown in Table 1. Overall, elderly patients were more likely to be female, white, live in metropolitan areas, have non-private insurance, have stage IIIA disease, and have more comorbidities compared to the non-elderly. Elderly patients had lower income and were less likely to be treated in academic centers.

**Table 1:** Patient characteristics in the elderly and non-elderly cohorts

Characteristic	Elderly (*N* = 57602)	Non-Elderly (*N* = 55928)	*P*-value
Age, mean (SD), years	77.5 (5.3)	60.2 (7.0)	N/A
Gender			< 0.0001
Female	26217 (45.5)	24050 (43.0)	
Male	31385 (54.5)	31878 (57.0)	
Race			< 0.0001
White	50439 (87.6)	45708 (81.7)	
Non-white	7163 (12.4)	10220 (18.3)	
Charlson-Deyo score			< 0.0001
0	33237 (57.7)	35177 (62.9)	
1–2	24365 (42.3)	20751 (37.1)	
Median income			< 0.0001
≥$48,000	26397 (45.8)	28604 (51.1)	
<$48,000	31205 (54.2)	27324 (48.9)	
Primary payor			< 0.0001
Private	5562 (9.7)	25033 (44.8)	
Non-private	52040 (90.3)	30895 (55.2)	
County Location			< 0.0001
Metropolitan	47064 (81.7)	44545 (79.7)	
Non-metropolitan	10538 (18.3)	11383 (20.3)	
Distance to closest facility, mean (SD), miles	20.2 (78.4)	23.0 (81.0)	< 0.0001
Facility type			< 0.0001
Academic	14682 (25.5)	16929 (30.3)	
Non-academic	42920 (74.5)	38999 (69.7)	
Clinical stage group			< 0.0001
IIIA	31973 (55.5)	27974 (50.0)	
IIIB	25629 (44.5)	27954 (50.0)	

*Abbreviation*: *SD* standard deviation

### Patterns of care

**Fig. 2:**
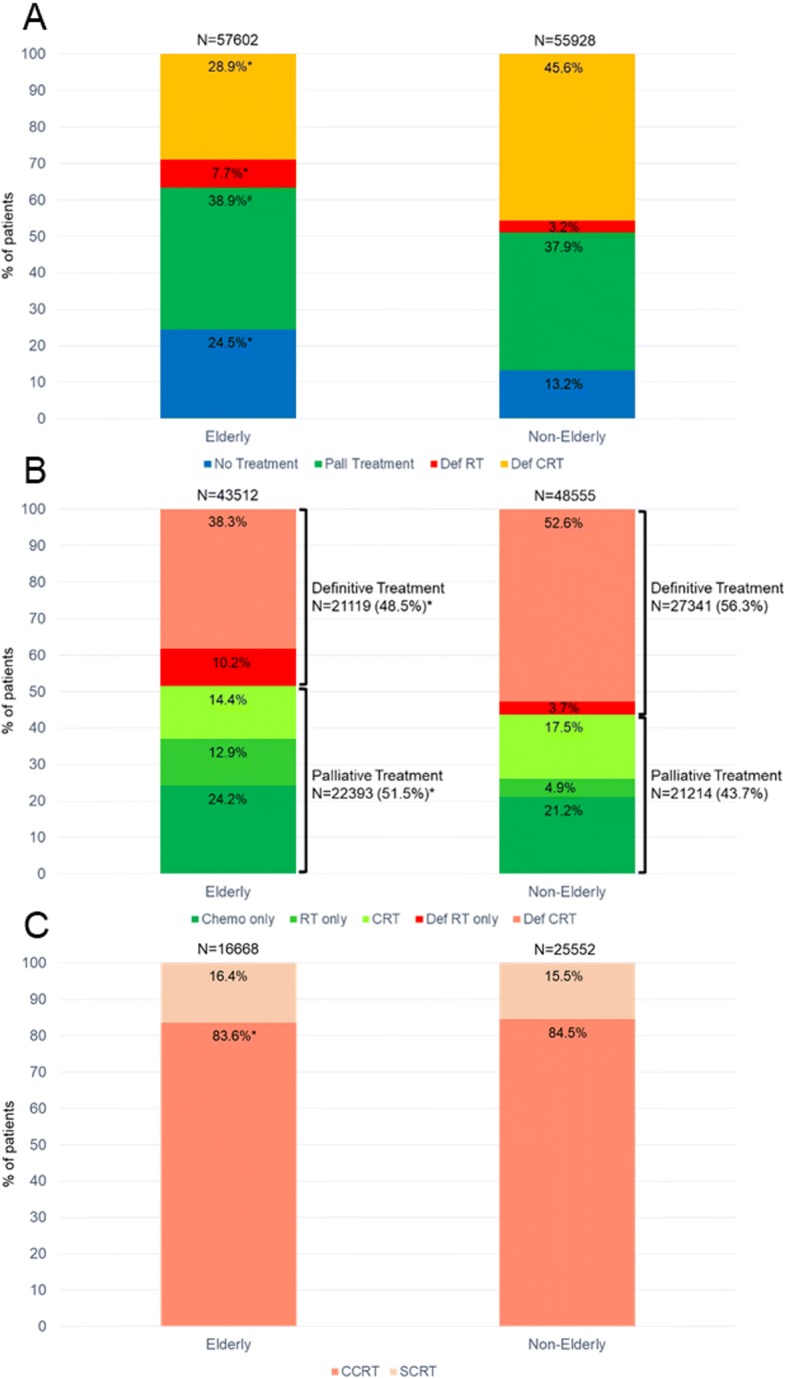
Bar graphs representing key comparisons of treatment patterns in the elderly vs. non-elderly patients: (**a**) Proportion of all patients included in the study who received no treatment, palliative (Pall) treatment, definitive radiation therapy (Def RT), and definitive chemoradiation (Def CRT), *P < 0.0001, #*P* = 0.0005. (**b**) Proportion of patients receiving any treatment further subdivided into definitive (Def RT only – definitive radiation therapy alone, Def CRT – definitive chemoradiation including sequential and concurrent chemoradiation), and palliative treatments (Chemo only – chemotherapy alone, RT only – palliative radiation therapy alone, CRT – palliative chemoradiation), *P < 0.0001. (**c**) Proportion of patients receiving definitive chemoradiation who received sequential (SCRT) and concurrent (CCRT) chemoradiation, *P = 0.016

Figure 2a provides an overall summary of treatments administered to the patients included in this study. When including the 57,602 elderly and 55,928 non-elderly patients, a significantly higher proportion of elderly patients received no treatment (24.5% vs. 13.2%, *P* < 0.0001), palliative treatment (38.9% vs. 37.9%, *P* = 0.0005), and definitive radiation therapy alone (7.7% vs. 3.2%, *P* < 0.0001), and a lower proportion received definitive chemoradiation (28.9% vs. 45.6%, *P* < 0.0001).

#### No treatment vs. treatment

As seen in Fig. 2a, the elderly had a significantly higher proportion of patients that did not receive any treatment compared to the non-elderly: 24.5% vs. 13.2% (*P <* 0.0001). On multivariate analysis (Table 2), elderly patients were nearly twice as likely to not receive treatment: OR = 1.97 (95% CI 1.90–2.03, *P <* 0.0001). Other factors associated with increased odds of not receiving treatment included patients with any comorbidities (OR = 1.33, 95% CI 1.29–1.37), female patients (OR = 1.13, 95% CI 1.09–1.16), and patients with higher income (OR = 1.06, 95% CI 1.03–1.10).

**Table 2:** Logistic regression analysis of factors associated with not receiving treatment (OR > 1 means first factor is associated with a higher odds of not receiving treatment)

No Treatment vs. Treatment	Univariate Analysis			Multivariate Analysis		
Variable	OR	95% CI	*P*-value	OR	95% CI	*P*-value
Elderly vs. Non-Elderly	2.13	2.07–2.20	< 0.0001	1.97	1.90–2.03	< 0.0001
Female vs. male	1.13	1.10–1.17	< 0.0001	1.13	1.09–1.16	< 0.0001
White vs. non-white	0.91	0.87–0.95	< 0.0001	0.84	0.80–0.87	< 0.0001
Academic vs. non-academic	0.93	0.90–0.96	< 0.0001	0.98	0.94–1.01	0.1751
Private vs. non-private insurance	0.56	0.54–0.58	< 0.0001	0.77	0.75–0.81	< 0.0001
Median income (≥$48,000 vs. <$48,000)	1.07	1.04–1.10	< 0.0001	1.06	1.03–1.10	0.0002
County location (Metropolitan vs. non-metropolitan)	1.03	0.99–1.07	0.1080	–	–	–
Clinical stage IIIB vs. stage IIIA	0.97	0.94–1.00	0.0406	1.02	0.99–1.05	0.2312
Distance to closest facility^a^	0.98	0.97–0.99	0.0006	1.01	1.00–1.02	0.1479
Charlson-Deyo score (1 vs. 0)	1.40	1.35–1.44	< 0.0001	1.33	1.29–1.37	< 0.0001

*Abbreviations*: *CI* confidence interval, *OR* odds ratio; ^a^log(distance) was used for analysis

#### Palliative treatment vs. definitive treatment

Amongst the 43,512 elderly and 48,555 non-elderly patients that received any treatment, Fig. 2b shows that the elderly were most likely to be treated with palliative intent: 51.5% vs. 43.7% (*P <* 0.0001). On multivariate analysis (Table 3), this translated into the elderly having a 38% increased odds of receiving palliative treatment (OR = 1.38, 95% 1.34–1.42). Stage IIIB (vs. stage IIIA) was associated with the highest odds of receiving palliative vs. definitive treatment (OR = 1.80, 95% CI 1.75–1.84). Other factors associated with higher odds of receiving palliative treatment included female gender, metropolitan county location, and more comorbidities while white patients and those with private insurance were more likely to receive definitive treatment.

**Table 3:** Logistic regression analysis of factors associated with palliative treatment amongst patients that received any treatment (OR > 1 means that the first factor is associated with higher odds of receiving palliative treatment)

Palliative vs. Definitive Treatment	Univariate Analysis			Multivariate Analysis		
Variable	OR	95% CI	*P*-value	OR	95% CI	*P*-value
Elderly vs. Non-Elderly	1.37	1.33–1.40	< 0.0001	1.38	1.34–1.42	< 0.0001
Female vs. male	1.10	1.07–1.13	< 0.0001	1.11	1.08–1.14	< 0.0001
White vs. non-white	0.93	0.90–0.97	0.0001	0.91	0.88–0.95	< 0.0001
Academic vs. non-academic	1.01	0.98–1.04	0.4758	–	–	–
Private vs. non-private insurance	0.83	0.80–0.85	< 0.0001	0.92	0.89–0.95	< 0.0001
Median income (≥$48,000 vs. <$48,000)	0.95	0.93–0.98	0.0002	0.98	0.95–1.00	0.0762
County location (Metropolitan vs. non-metropolitan)	1.12	1.08–1.15	< 0.0001	1.11	1.07–1.16	< 0.0001
Clinical stage IIIB vs. stage IIIA	1.73	1.69–1.78	< 0.0001	1.80	1.75–1.84	< 0.0001
Distance to closest facility^a^	0.98	0.97–0.99	0.0006	1.01	1.00–1.02	0.0598
Charlson-Deyo score (1 vs. 0)	1.19	1.16–1.23	< 0.0001	1.20	1.17–1.23	< 0.0001

*Abbreviations*: *CI* confidence interval, *OR* odds ratio; ^a^log(distance) was used for analysis

For the patients treated with palliative intent who received radiation therapy, the radiation doses were further binned based on dose. Of the 22,726 patients who received radiation as part of their palliative treatment, 6298 (27.7%) received a dose >8 and ≤30 Gy, 7,708 (33.9%) received >30 and ≤45 Gy, and 8,720 (38.4%) received >45 and <59.4 Gy. The median biological effective dose (using α/β = 10) based on the linear quadratic formula for this group was 63.4 Gy_10_ (IQR = 59.5–67.6 Gy_10_) and the median fraction size was 2 Gy (IQR, 1.8–2.25 Gy).

#### Definitive treatment: RT alone vs. CRT

Within the group of 21,119 elderly and 27,341 non-elderly patients treated with definitive therapy, a significantly higher proportion of the elderly were treated with RT alone (10.2% vs. 3.7%, *P <* 0.0001, Fig. 2b), which resulted in a >3-fold increase in the likelihood of receiving RT alone for the elderly (OR = 3.30, 95% CI 3.10–3.51) on multivariate analysis (Table 4). Patients with private insurance were 32% less likely to receive RT alone (OR = 0.68, 95% CI 0.63–0.73). Other factors resulted in modest increases (more comorbidities, higher income) or decreases (stage IIIB, further distance to treatment facility) in the likelihood of patients receiving RT alone.

**Table 4:** Logistic regression analysis of receiving radiation therapy alone versus chemoradiation in patients that received definitive treatment (OR > 1 means that first factor is associated with higher odds of radiation therapy alone)

Radiation Therapy Alone vs. Chemoradiation	Univariate Analysis			Multivariate Analysis		
Variable	OR	95% CI	*P*-value	OR	95% CI	*P*-value
Elderly vs. Non-Elderly	3.81	3.30–4.04	< 0.0001	3.30	3.07–3.51	< 0.0001
Female vs. male	1.05	0.99–1.11	0.0840	1.04	0.99–1.10	0.1445
White vs. non-white	0.96	0.89–1.03	0.2684	–	–	–
Academic vs. non-academic	0.91	0.86–0.97	0.0024	1.02	0.96–1.09	0.5296
Private vs. non-private insurance	0.38	0.35–0.41	< 0.0001	0.68	0.63–0.73	< 0.0001
Median income (≥$48,000 vs. <$48,000)	1.18	1.12–1.25	0.0002	1.26	1.19–1.33	< 0.0001
County location (Metropolitan vs. non-metropolitan)	1.07	1.00–1.15	0.0473	1.05	0.97–1.14	0.2267
Clinical stage IIIB vs. stage IIIA	0.73	0.69–0.77	< 0.0001	0.82	0.78–0.87	< 0.0001
Distance to closest facility^a^	0.90	0.88–0.92	< 0.0001	0.93	0.91–0.95	< 0.0001
Charlson-Deyo score (1 vs. 0)	1.31	1.24–1.38	< 0.0001	1.22	1.15–1.29	< 0.0001

*Abbreviations*: *CI* confidence interval, *OR* odds ratio; ^a^log(distance) was used for analysis

Analyzing only patients that received CRT (*N* = 16,668 elderly and 25,552 non-elderly), a higher proportion of elderly patients received SCRT compared to the non-elderly (16.4% vs. 15.5%, *P* = 0.016) as seen in Fig. 2c. In total, 13,940 elderly patients and 21,594 non-elderly patients received CCRT. The respective differences in proportions of elderly patients vs. non-elderly patients that received CCRT when looking at the entire population, only patients that received treatment, and only patients that received definitive treatment were: 24.2% vs. 38.6% (*P <* 0.0001); 32.0% vs. 44.5% (*P <* 0.0001); 66.0% vs. 78.9% (*P <* 0.0001).

When CRT was delivered, most patients received a multi-agent chemotherapy vs. single-agent chemotherapy regimen. However, a lower proportion of elderly patients received multi-agent chemotherapy compared to the non-elderly: 91.8% vs. 95.3% in the CCRT patients (*P <* 0.0001), and 91.1% vs. 96.1% (*P <* 0.0001) in the SCRT patients. For the 48,460 patients treated with definitive intent, 29,233 (60.3%) received a dose ≥59.4 Gy and <66 Gy, 13,821 (28.5%) received ≥66 Gy and <70 Gy, and 5,406 (11.2%) received doses ≥70 Gy.

Given the wide range of palliative radiation therapy doses used, we performed a sensitivity analysis where the 8,720 patients who received >45 and <59.4 Gy were included as part of the definitive rather than palliative treatment group. The results of this analysis are shown in Additional file 1: Figure S1. The overall results remained the same with the elderly more likely to not receive treatment (24.5% vs. 13.2%, *P* < 0.0001), more likely to receive a palliative treatment (31.3% vs. 30.1%, *P* < 0.0001), and more likely to receive definitive RT alone (10.7% vs. 4.4%, *P* < 0.0001) than definitive CRT (33.5% vs. 52.3%, *P* < 0.0001) when compared to non-elderly patients.

## Discussion

In summary, we found substantial disparities in the management of non-surgically treated stage III NSCLC based on age (≥70 years old vs. <70 years old) in the largest study to date. Compared to non-elderly patients, elderly patients were twice as likely to not receive any treatment and 1.4 times more likely to receive palliative treatment when treatment was delivered. When definitive treatment was delivered, the elderly were > 3-fold more likely to receive radiation therapy alone as opposed to CRT. Of note, in the subset of patients treated with definitive CRT, there was little absolute difference observed between the elderly and non-elderly in those that received SCRT (16.4% versus 15.5%). While this 0.9% difference was statistically significant, a larger absolute difference in the SCRT rates between elderly and non-elderly patients may have been expected. However, the smaller difference in SCRT rates is undoubtedly impacted by the overall key finding of our study which is that a significantly smaller proportion of elderly patients receive definitive CRT compared to the non-elderly.

Consistent with other reports, a large proportion of elderly patients with locally advanced NSCLC did not receive any type of treatment [8, 19]. In our patient cohort, 24.5% of elderly patients did not receive treatment which was significantly higher than the proportion of patients <70 years old (13.2%). In a SEER-Medicare analysis of 6,325 patients >65 years old with locally advanced NSCLC, 26.5% of patients did not receive any cancer directed treatment [8]. Wang et al. utilized the Veterans Affairs Central Cancer Registry to evaluate the administration of guideline recommended treatment in patients ≥65 years old with NSCLC [19]. Older patients with no comorbidities were administered guideline recommended treatment less often than younger patients with severe comorbidities. Similarly, we found that when treatment was administered, fewer than one-third of the elderly patients received definitive CRT compared to nearly half of the non-elderly patients.

Recently, Davidoff et al. used the NCDB to study patterns of care and outcomes in 12,641 octagenarians and nonagenerians with stage III NSCLC [8]. These patients were categorized into one of 3 groups: no treatment, RT alone (≥45 Gy), or CRT. In this subset of elderly patients with advanced age, more than 60% did not receive any treatment at all. While the definition of treatment was not exactly the same as the one used in our study, we both found similar factors associated with patients not receiving treatment: increasing age, more comorbidities, non-white race, and female sex.

In a population-based dataset from the Netherlands Cancer Registry, Driessen et al. examined treatment patterns and survival in older patients (65–74 years old, *N* = 3,876 and ≥75 years old, *N* = 3,163) with stage III NSCLC from 2009 to 2013 [20]. In this dataset, 17% of patients aged 65–74 years old and 39% of patients aged ≥75 years did not receive any treatment for a total of approximately 27% of patients ≥65 years old not receiving any treatment. This rate is remarkably similar to the 24% of patients ≥70 years old in the United States NCDB that did not receive any treatment.

The same group has also examined changes in treatment patterns for all stages of NSCLC in patients <70 years old and ≥70 years old from 1990 to 2014 [21]. In both younger patients and older patients, the proportion that do not receive treatment has declined over time from 18% (1990–1994) to 10% (2010–2014) in younger patients and from 32% (1990–1994) to 29% (2010–2014) in the older patients. In the most recent cohort (2010–2014), the proportion of younger patients receiving CRT was 48% which is comparable to the 46% of younger patients in our study that received CRT. Similarly, 27% of elderly patients received CRT in the Netherlands Cancer Registry compared to 29% in our study.

These cohort studies demonstrate that elderly patients with stage III NSCLC are less likely to receive standard curative treatments compared to younger patients. CRT for stage III NSCLC, particularly CCRT, can result in severe acute morbidities including esophagitis, hematologic toxicity, and pneumonitis, particularly in elderly patients [4, 9–11, 13, 22–27]. Furthermore, CCRT is associated with ~ 2% risk of treatment-related acute mortality in contemporary phase III trials [28, 29]. The morbidity/mortality concerns most likely factor into the treatment decision for elderly patients. In a systematic review of the use of a comprehensive geriatric assessment (CGA) tool in elderly patients with NSCLC, Schulkes et al. found that the CGA can help detect several health concerns not reflected by the patient’s Eastern Cooperative Oncology Group performance status and that certain CGA domains are predictive of mortality and treatment completion [30]. In order to help identify elderly patients that are at particularly high risk of treatment-related morbidity/mortality, use of a CGA is now endorsed by the National Cancer Network, the International Society for Geriatric Oncology, and the European Organization for the Research and Treatment of Cancer [31–33]. The CGA has not been a component of previous randomized studies in stage III NSCLC, but it should be incorporated in elderly patients treated on future trials to help validate its usefulness.

In addition to assessment tools that may help identify patients at higher risk of toxicity, there have also been demonstrable improvements in radiation therapy delivery that may help reduce toxicity and potentially improve survival in patients receiving CCRT for stage III NSCLC. In an NCDB analysis of patients with stage III NSCLC treated between 2003 and 2005, Sher et al. demonstrated that conformal radiotherapy techniques using computed-tomography (CT) based planning, including 3D conformal radiation therapy (3DCRT) and intensity-modulated radiation therapy (IMRT), resulted in a statistically significant 3% absolute increase in 3-year and 5-year survival rates compared with conventional 2D radiation therapy [34]. Subsequently, Koshy et al. compared survival in patients with stage III NSCLC treated with IMRT vs. non-IMRT techniques from 2003 to 2011 in the NCDB. In this analysis, patients treated with IMRT had an 11% relative reduction in the risk of death, translating into an improved median survival time of 20 months vs. 18.2 months [35]. Furthermore, IMRT resulted in a lower incidence of radiation therapy treatment interruptions. With regards to toxicity, a secondary analysis of Radiation Therapy Oncology Group 0617, a randomized study of CCRT to 60 Gy vs. 74 Gy in unresectable stage III NSCLC, demonstrated that IMRT was associated with lower rates of severe radiation pneumonitis and resulted in overall lower cardiac doses when compared to patients treated with 3DCRT [36]. Taken together, these three studies conducted in a relatively modern treatment era suggest that CT based planning using IMRT has led to reduced morbidity and mortality compared to outdated radiation techniques – therefore, elderly patients treated with the modern radiation techniques currently available may be able to better tolerate aggressive treatment approaches such as CCRT.

Limitations of this study include its retrospective nature and the lack of toxicity data, which is not captured by the NCDB. In addition, while the NCDB captures the Charlson-Deyo score, it does not include other critical data elements such as performance status, smoking history, extent of disease radiographically, and patient preferences, all of which factor into the treatment-decision making process. Studies that use the NCDB to compare survival amongst various treatment groups are often appropriately criticized because despite the large sample size, it can be argued that the non-randomized retrospective nature of these studies actually amplifies selection bias and leads to equally amplified differences in survival between treatment groups. However, in this study, we present a patterns-of-care analysis, and not a comparison of survival amongst various treatment groups. In addition, we may be criticized for the decision to use ≥59.4 Gy as the radiation dose that defines definitive intent. Alternative fractionation schedules with doses that approach an equivalent dose of near 60 Gy in 30 fractions (continuous hyperfractionated accelerated radiation therapy, hypofractionated radiation (≥3 Gy/fraction) schedules to 50–60 Gy total dose, etc.) are the subject of much discussion for patients that cannot tolerate concurrent chemotherapy. In order to account for the possibility that the patients classified as having received palliative radiation therapy to doses of >45 Gy but <59.4 Gy may have received definitive treatment, we performed a sensitivity analysis in which all of these patients were classified as having received definitive radiation therapy. Our results did not change, which solidified our decision for 59.4 Gy as the cutoff for distinguishing definitive vs. palliative radiation therapy. Ultimately, shorter hypofractionated schedules may be the best approach for elderly patients, and we eagerly await the results of a current randomized phase III trial testing 60 Gy in 15 fractions to 60 Gy in 30 fractions [37].

Despite these limitations, our study has notable strengths. First, we present a detailed patterns of care analysis that characterizes the treatment of ~70% of every non-surgically treated stage III NSCLC in the United States. In effect, we have described the actual treatment delivered to the vast majority of patients in academic and non-academic centers in the U.S. Moreover, our analysis includes a rigorous assessment of how each patient was treated, especially with regard to radiation dose and target volume, which are details that are not available in the SEER database. To date, this is the largest (57,602 elderly patients and 55,928 non-elderly patients) and most comprehensive analysis highlighting the disparities in patterns of care in elderly vs. non-elderly patients with non-surgically treated stage III NSCLC. Given the recent results of the PACIFIC trial, which showed a progression-free survival benefit for patients with locally advanced, unresectable stage III NSCLC without disease progression after chemoradiation, it will be interesting to see how the use of immunotherapy impacts patterns of care in the elderly and non-elderly in the next 5–10 years [38].

## Conclusions

We found that elderly patients with non-surgically treated stage III NSCLC are less likely to receive any treatment, definitive treatment, and CRT compared to non-elderly patients. As a whole, standard of care CCRT was administered to <25% of all elderly patients. Even when a definitive treatment course was delivered, more than 20% of elderly patients received RT alone. The lack of use of concurrent or sequential chemotherapy in the elderly with stage III NSCLC suggests that the optimal treatment approach for this vulnerable population remains undefined.

## Additional file

Additional file 1: **Figure S1.** Sensitivity analysis for all patients included in the study who received no treatment, palliative (Pall) treatment, definitive radiation therapy (Def RT), and definitive chemoradiation (Def CRT) including the 8,720 patients who received >45 and <59.4 Gy as part of the definitive rather than palliative treatment group. The overall results remained the same, *P < 0.0001. (TIFF 3486 kb)

## Abbreviations

3DCRT: 3D conformal radiation therapy
AJCC: American joint committee on cancer
CCRT: Concurrent chemoradiation
CGA: Comprehensive geriatric assessment
COC: Commission on cancer
CRT: Chemoradiation
CT: Computed-tomography
IGRT: Image-guided radiation therapy
IMRT: Intensity-modulated radiation therapy
NCDB: National cancer database
NSCLC: Non-small cell lung cancer
RT: Radiation therapy
SCRT: Sequential chemoradiation
