# Supplementary figures and images for: Identifying patterns of care for elderly patients with non-surgically treated stage III non-small cell lung cancer: an analysis of the national cancer database
Source: Radiat Oncol. 2018 Oct 5;13:196. doi: 10.1186/s13014-018-1142-7 (PMC6173899; doi:10.1186/s13014-018-1142-7)

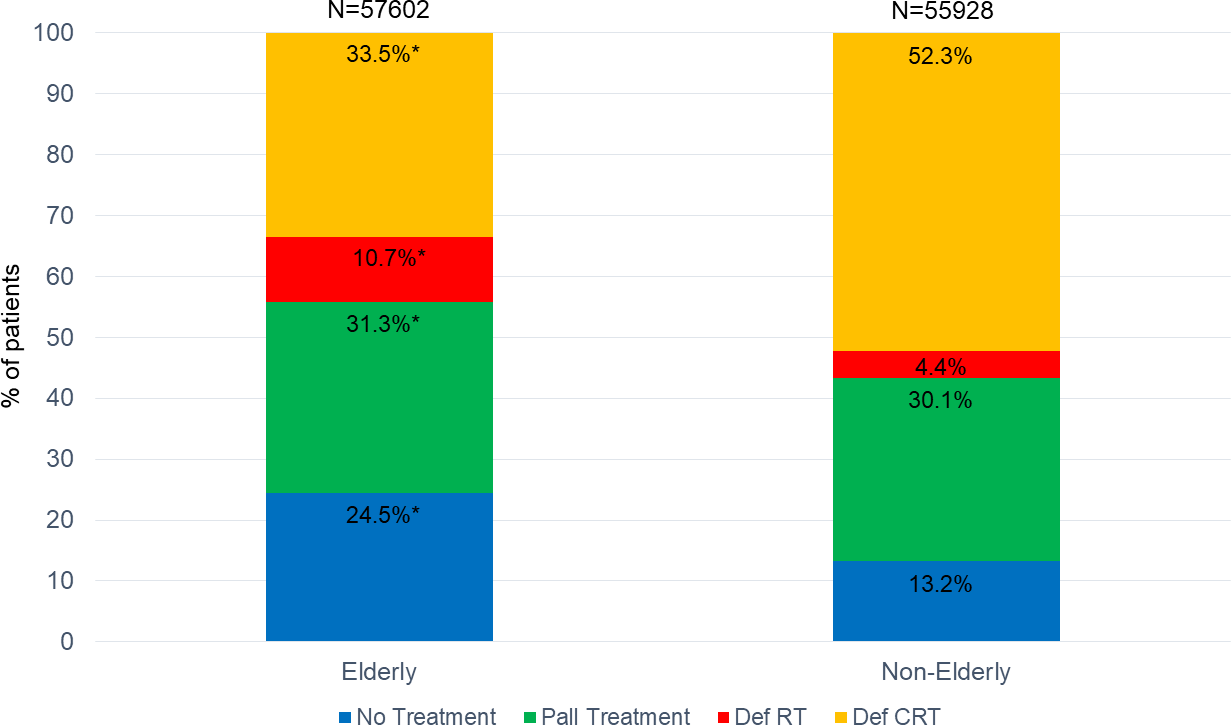

Supplement: Supplementary file 1 — Figure S1. Sensitivity analysis for all patients included in the study who received no treatment, palliative (Pall) treatment, definitive radiation therapy (Def RT), and definitive chemoradiation (Def CRT) including the 8,720 patients who received >45 and <59.4 Gy as part of the definitive rather than palliative treatment group. The overall results remained the same, *P < 0.0001. (TIFF 3486 kb) [file 13014_2018_1142_MOESM1_ESM.tiff]
